# Variations of main quality components of matcha from different regions in the Chinese market

**DOI:** 10.3389/fnut.2023.1153983

**Published:** 2023-03-09

**Authors:** Ying Luo, Yazhao Zhang, Fengfeng Qu, Wenjun Qian, Peiqiang Wang, Xuzhou Zhang, Xinfu Zhang, Jianhui Hu

**Affiliations:** ^1^College of Horticulture, Qingdao Agricultural University, Qingdao, China; ^2^Qingdao Technical College, Qingdao, China

**Keywords:** matcha, tea polyphenol, theanine, volatile components, solvent-assisted flavor evaporation, headspace solid-phase microextraction

## Abstract

Matcha has a unique aroma of seaweed-like, which is popular with Chinese consumers. In order to effectively understand and use matcha for drinks and tea products, we roundly analyzed the variation of main quality components of 11 matcha samples from different regions in the Chinese market. Most of matcha samples had lower ratio of tea polyphenols to amino acids (RTA), and the RTA of 9 samples of matcha was less than 10, which is beneficial to the formation of fresh and mellow taste of matcha. The total volatile compounds concentrations by HS-SPME were 1563.59 ~ 2754.09 mg/L, among which terpenoids, esters and alcohols were the top three volatile components. The total volatile compounds concentrations by SAFE was 1009.21 ~ 1661.98 mg/L, among which terpenoids, heterocyclic compounds and esters ranked the top three. The 147 volatile components with high concentration (>1 mg/L) and no difference between samples are the common odorants to the 11 samples of matcha. The 108 distinct odorants had differences among the matcha samples, which were important substances leading to the different aroma characteristics. Hierarchical cluster analysis (HCA) and orthogonal partial least squares discriminant analysis (OPLS-DA) showed that 11 samples of matcha were well clustered according to different components. Japanese matcha (MT, MY, ML, MR, MJ) could be clustered into two categories. The aroma composition of Guizhou matcha (GM1, GM2) was similar to that of Japanese matcha, 45 volatile components (decanal, pyrazine, 3,5-diethyl-2-methyl-, 1-hexadecanol, etc. were its characteristic aroma components. The aroma characteristics of Shandong matcha and Japanese matcha (ML, MR, MJ) were similar, 15 volatile components (γ-terpinene, myrtenol, *cis*-3-hexenyl valerate, etc.) were its characteristic aroma components. While Jiangsu matcha and Zhejiang matcha have similar aroma characteristics due to 225 characteristic aroma components (coumarin, furan, 2-pentyl-, etc). In short, the difference of volatile components formed the regional flavor characteristics of matcha. This study clarified the compound basis of the flavor difference of matcha from different regions in the Chinese market, and provided a theoretical basis for the selection and application of matcha in drinks and tea products.

## Introduction

Due to the unique green aroma with some sweet and roasted odors (Seaweed-like), expanded food processing characteristics and people’s demand for health products, matcha is popular in China and even the world (1–3). At present, China’s matcha production accounts for about 60% of the world’s total, mainly distributed in Zhejiang province, Jiangsu province, Guizhou province and other places. Moreover, with the diversification and high-grade development of matcha products and the expansion of matcha consumer groups, the matcha industry has great development potential.

As we all know, the flavor quality of matcha is mainly reflected in the aroma and taste. Previous studies on the chemical constituents and sensory properties of green tea (4), black tea (5) and white tea (6) have shown that the chemical constituents closely related to taste include free amino acids, tea polyphenols, L-theanine and so on. The production of matcha has a unique shading technology, which can effectively reduce caffeine, tea polyphenols and other astringent ingredients in fresh leaves, effectively increase the content of amino acids, improve the freshness of the taste of matcha, increase the content of chlorophyll, and improve the sensory quality of matcha (7, 8).

As one of the most important determinants of tea quality, aroma is mainly composed of volatile components. Although it only accounts for 0.01% ~ 0.05% in tea, the different composition and proportion of these volatile compounds in tea affect the concentration and flavor type of tea aroma. Matcha has a unique and pleasant aroma quality, which is deeply loved by tea consumers. Compared with green tea, the aroma of matcha is fresher. Through the analysis of hexane extract in tencha (raw material of matcha) by gas chromatography-olfactory method (GC-O), the predecessors found that tencha has a unique seaweed-like aroma (9). Recent studies have found that tencha-ro is the best method for producing high quality tencha with a strong seaweed-like aroma, and identified 2-ethyl-3,5-dimethylpyrazine, 2-ethyl-6-methylpyrazine, 2-ethyl-5-methylpyrazine, dimethyl sulfide, β-ionone, and 2-formyl-1-methylpyrrole is an important contribution to the seaweed-like aroma of tencha (10). 8 compounds such as coumarin, α-ionone, (Z)-1, 5-octadien-3-one, 4-hydroxy-2,5-dimethyl-3(2H)-furanone, were the key to the aroma of Japanese matcha (11). Our previous study showed that the characteristic volatile components of high-grade matcha include 3-methyl-2-butene-1-thiol, 3-ethyl-phenol, 2-thiophenemethanethiol and so on. After grinding tea into matcha, the content of volatile components was increased, and the aroma and flavor characteristics of matcha were enhanced (12). At present, there are many kinds of products from different regions in the Chinese market, in order to deepen the understanding of the aroma of matcha, clarifying the flavor characteristics of matcha of different regions, and characterizing its volatile components have important value.

The flavor of matcha could be affected by the tea cultivars, cultivation measures, product grade and other factors (11–13). For example, the effects of tea clone, shading degree and shooting period on volatile components in Turkish green tea powder were compared. It was found that although there were some significant changes in the volatile compounds of green tea powder according to the level of shading treatment, there was no significant difference in the volatile compounds of samples in the overall evaluation (14). Previous research reported that *trans*-4,5-epoxy-(E)- 2-decenal, coumarin and other substances are the key aroma components in matcha by using gas chromatography-olfactometer (GC-O) (11). Researchers compared and characterized volatiles of different Japanese green teas (sencha, matcha, and hojicha), and found that nonanal is an important aroma compound of matcha, and steamed green tea contains higher flower fragrance (13). Regional factors are also important factors that affect the flavor of matcha. There are relatively few studies on the analysis and comparison of the flavor differences of matcha between different regions.

Currently, the methods for extracting volatile components from tea include headspace solid phase microextraction (HS-SPME) (15), solvent-assisted flavor evaporation (SAFE) (12, 13), simultaneous-distextraction (SDE) (16) and so on. Among them, HS-SPME has the advantages of simple and rapid sample preparation, and can be highly automated, it has a series of single or multi-fiber phases with different polarity and molecular size, which is helpful to effectively extract the target analytes (17). SAFE is a method that enables the separation of volatile compounds from complex substrates in a cryogenic vacuum environment (18). However, HS-SPME is not effective in extracting compounds with low volatility (19), it can effectively extract compounds with high volatility, while SAFE has poor performance in extracting these compounds, because they are easily dissipated in the concentration process (19). Therefore, the combination of the two methods helps to obtain more comprehensive volatile components. Researchers used HS-SPME and SAFE to extract volatile compounds from Japanese matcha and found that they were complementary (13), which was also confirmed by our previous research (12). Therefore, in this study, we combined HS-SPME with SAFE to obtain more comprehensive volatile components in matcha from different regions.

In order to obtain representative samples of matcha, we first collected 25 samples of matcha from different producing areas for sensory evaluation, and then selected 11 matcha samples with seaweed-like aroma characteristics for quality components analysis. The quality analysis in this study mainly involves the microscopic morphology, color, basic taste components and comprehensive volatile aroma components of matcha. PCA analysis, HCA analysis and OPLS-DA analysis were used to clarify the aroma characteristics of matcha for revealing the differences of volatile components among matcha in different regions. The results obtained from this study will provide a theoretical basis for the selection and application of matcha in drinks and tea products.

## Materials and methods

### Matcha samples

A total of 25 matcha samples were collected from different tea producing areas. In order to select representative samples of matcha, we took the national standards, local standards and group standards of matcha as the basis for screening. Only those samples that meet the sensory quality requirements of matcha selected, and all of them present different degrees of seaweed-like fragrance, either rich or slight. These standards include Matcha (GB/T 34778–2017), Guizhou Matcha (DB52/T 1358–2018), Technical specification of matcha sensor evaluation (DB33/T 2279–2020) and Shandong Matcha (T/SDAS 192–2020). The specific information of the matcha samples is shown in table (Table 1). Japanese matcha (MT, MY, ML, MR, and MJ) from Japan, Guizhou matcha (GM1, GM2), Zhejiang matcha (ZM1, ZM2), Jiangsu matcha (JM) and Shandong matcha (M-GS) were from Guizhou province, Zhejiang province, Jiangsu province and Shandong province of China, respectively.

**Table 1:** The sample information of matcha with seaweed-like odor.

Samples	Odor description	Region	Manufacturer
MT	Rich seaweed-like odor	Japanese matcha	Yuji Matcha (Shanghai) Co., Ltd
MY	Rich seaweed-like odor	Japanese matcha	Yuji Matcha (Shanghai) Co., Ltd
ML	Medium seaweed-like odor	Japanese matcha	Yuji Matcha (Shanghai) Co., Ltd
MR	Medium seaweed-like odor	Japanese matcha	Yuji Matcha (Shanghai) Co., Ltd
MJ	Medium seaweed-like odor	Japanese matcha	Yuji Matcha (Shanghai) Co., Ltd
GM1	Rich seaweed-like odor	Guizhou matcha	Guizhou Guicha (Group) Co., Ltd
GM2	Medium seaweed-like odor	Guizhou matcha	Guizhou Guicha (Group) Co., Ltd
ZM1	Medium seaweed-like odor	Zhejiang matcha	Hangzhou Yuchacun Tea Industry Co., Ltd
ZM2	Little seaweed-like odor	Zhejiang matcha	Hangzhou Yuchacun Tea Industry Co., Ltd
JM	Medium seaweed-like odor	Jiangsu matcha	Jiangsu Xinpin Tea Co., Ltd
M-GS	Medium seaweed-like odor	Shandong matcha	Qingdao Hongyu Matcha Technology Development Co., Ltd

### Observation on the appearance and morphology of matcha

#### Microscopic morphology observation

Matcha powder from different regions were sprinkled on the double-sided conductive adhesive, sprayed with ion sputtering instrument, and placed under a scanning electron microscope (Japan Electron JSM-7500F03040702) to a size of 1,000 times for observation and photography.

#### Determination of color and chlorophyll content

Color difference analysis: Weighed each matcha sample 5.0 g and placed it on the calibrated color difference meter, calibrated it with the white board for measurement, recorded the L value (brightness), a value (red and green degree), and b value (yellow and blue degree), and repeated the measurement for 3 times for each sample.

##### Chlorophyll content determination

The method of reference (20) was followed. Weighed 0.04 g sample and placed it in a volumetric bottle, added 6 mL 80% acetone solution, placed it in dark place at room temperature for extraction overnight, shaked it three times during the period. After 14 h, 80% acetone solution was filled to 10 mL and centrifugation was performed for colorimetry. The absorbance of chlorophyll a and b was determined at the wavelength of 663 nm and 645 nm, respectively. The total chlorophyll content was the sum of the content of chlorophyll a and b.

### Determination of basic quality components contributing to taste

The contents of tea polyphenols, L-theanine, free amino acid and caffeine were determined according to Chinese standard GB/T 8313–2018, GB/T 23193–2017, GB/T 8314–2013 and GB/T 8312-2013respectively. The content of soluble sugar was detected using the plant soluble sugar kit (Suzhou Keming Biotechnology Co., Ltd., Suzhou, China).

### Extraction and determination of volatile components

#### Headspace solid phase microextraction procedure

The method of reference (12) was followed with minor changes. 1.00 g of each sample was weighed into a headspace vial, and 3 mL saturated NaCl (Sinopharm Chemical Reagent Co., Ltd (Shanghai, China) solution and 10 μL (50 μg/mL) of internal standard solution containing (R)-(−)-Carvone-4,4,6,6-d4 (cdn, Quebec, Canada) were added, respectively. At a constant temperature of 100°C, the sample was oscillated for 5 min, and inserted the 50/30 μm DVB/CAR/PDMS extraction head into the head-space vial, headspace extraction for 15 min. The sample was analyzed for 5 min at 250°C, and then separated and identified by GC–MS. Before sampling, the extraction head was aged at 250°C in a Fiber Conditioning Station for 5 min.

#### Solvent-assisted flavor evaporation procedure

The method of reference (12) was followed with minor changes. The matcha samples were weighed 7.00 g and put into a 100 ml conical flask with a stopper, and added 70 mL of dichloromethane (Tianjin Kemiou Chemical Reagent Co., Ltd., Tianjin, China) for extraction for 17 h, the extraction was carried out at 4°C. After extraction, centrifuged at 4°C for 15 min (5,500 rpm) and filtered it. And 70 μL (86.50 mg/L) of internal standard solution containing ethyl decanoate (Shanghai Yuanye Bio-Technology Co., Ltd., Shanghai, China) was accurately added. The temperature of constant temperature water bath and circulating water was set to 40°C, liquid nitrogen was added into the cold trap and thermos flask, when the pressure of system dropped to 5 × 10^−3^ Pa, the solution was poured into the drop funnel. After that, the drop funnel was opened, allowing the tea solution to drop slowly and evenly into the distillation flask. After extraction, the extract in the receiving flask was concentrated to about 5 mL by rotary evaporation, finally, the extract was blown to 0.5 mL with nitrogen. The temperature was set at 60°C and the extraction head was 120 μm DVB/CWR/PDMS. The rest of the operation was consistent with HS-SPME.

#### GC–MS analysis

The technology we use was widely-targeted volatile method, and the method of reference (21) was followed with minor changes. DB-5MS capillary column (30 m × 0.25 mm × 0.25 μm, Agilent J&W Scientific, Folsom, CA, United States), the flow rate of GC carrier gas (helium) was 1.2 mL/min, the temperature of the injection port was 250°C, no shunt injection, and the solvent is delayed for 3.5 min. The heating program was as follows: the initial temperature was 40°C which was held for 3.5 min, the temperature was increased to 100°C at the rate of 10°C/min, and then to 180°C at the rate of 7°C/min, finally increased to 280°C at the rate of 25°C/min, and held thereat for 7 min (SAFE held thereat for 5 min).

Electron bombardment ion source (EI), an ion source temperature 230°C, a four-stage rod temperature of 150°C, a mass spectrum interface temperature of 280°C and an electron energy of 70 eV were used. The scan mode of HS-SPME and SAFE were selected ion detection mode (SIM), qualitative and quantitative ion accurate scanning.

#### Identification the of volatile component content

Volatile compounds were characterized by the National Institute of Standards and Technology (NIST) library search program. The relative concentration of volatile compounds is calculated as follows (12):

$$C_{i}=\frac{C_{is}\times A_{i}}{A_{is}}$$

𝐶𝑖 is any component (mg/L), the quality of the concentration 𝐶𝑖𝑠 is internal standard mass concentration (mg/L), the 𝐴𝑖 chromatographic peak area is arbitrary components and 𝐴𝑖𝑠 is the internal standard substance of chromatographic peak area.

#### Calculation of odor activity value

$$OAV=\frac{c}{OT}$$

where c is the concentration of the compound in the sample (mg/L) and OT is the odor threshold of the compound in water (mg/L). The OTs were taken from references (22, 23).

### Statistical analysis

Excel version 2020 (Microsoft, Redmond, United States) was used to sort out the experimental data, Metware Cloud platform (Metware, Wuhan, China) was used to screen the differential compounds,^1^ SIMCA version 14.1 (Umetrics, Sweden) was used for PCA, HCA and OPLS-DA analysis, and tree diagram was drawn. IBM SPSS version 20 (International Business Machines Corporation, Amonk, United States) was used for significance analysis, Graphpad prism version 8.0 (GraphPad Software, San Diego, United States) was drawn bar graph, and TB tools version 1.108 (Guangzhou, China) was used to draw cluster heat map.

## Results

### Analysis of appearance quality and chlorophyll content of matcha from different regions

**Figure 1:**
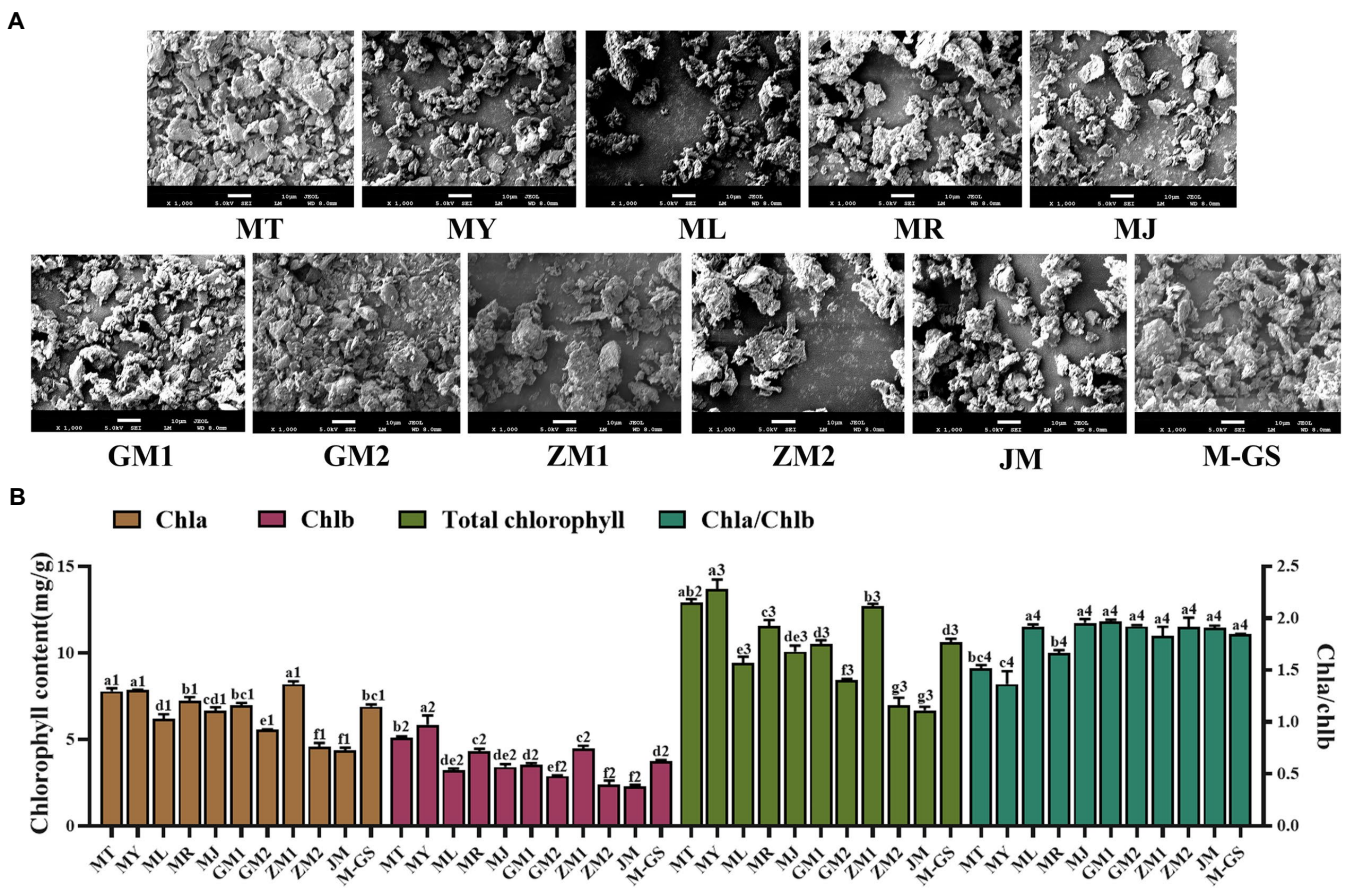
Scanning electron microscopy observation results and column chart of chlorophyll content of matcha from different regions. **(A)** Scanning electron microscopy observation results of matcha from different regions. **(B)** Column chart of chlorophyll content of matcha from different regions. The lowercase alphabetical letters on the column chart of different substances represent significant differences (*p*<0.05).

The results of scanning electron microscopy (Figure 1A) show that the particle size of MT, MY, JM and M-GS is with a diameter of about 3 ~ 10 μm, and other samples also meets the requirements of the national standard (≤ 18 μm). On the whole, the roundness of the surface of matcha is low and uneven. One of the reasons may be that the shear force of the tea sample is high during the grinding process, which leads to the uneven surface of the powder. Similar observations have been reported by the predecessors (24–26). The second reason is that the powder is not shaken and piled up.

The color of matcha is one of the important indicators to measure the quality of matcha. According to the result of color difference measurement value (Table 2). The highest brightness tea sample is GM2, followed by JM, GM1, MR and ML, while ZM1 and ZM2 is the lowest The ‘a’ value represents red-green degree, positive value is red degree, negative value is green degree. The lowest value of ‘a’ is MT, followed by GM1 and MY, while ZM2 has the largest a value. The ‘b’ value represents the yellow-blue degree, and the positive value is yellow degree, while the negative value is blue degree. The largest ‘b’ value is MY and GM1, followed by ML and MR, while ZM2 is the smallest. a/b represents the hue value, and its absolute value is proportional to the greenness of the tea sample. The order from highest to lowest in terms of hue value is MT > ZM1 > M-GS > GM1 > MY>MR > MJ > ML > GM2 > JM > ZM2. According to the results of chlorophyll composition and content (Figure 1B), the contents of chlorophyll a, chlorophyll b and total chlorophyll are higher in MT, MY, ZM1, MR and M-GS, while the contents of ZM2 and JM are the lowest. Researchers have proposed that the ratio of Chla to Chlb can be used to evaluate the quality of matcha (27), and the value was ranked in the order from highest to lowest GM1 >  MJ >  GM2 >  ZM2 >  ML >  JM >  M-GS >  ZM1 >  MR >  MT > MY. The ratio of all tea samples does not exceed 2, indicating that the color quality of matcha is superior.

**Table 2:** The color difference measurement value of matcha from different regions.

Samples	L	a	b	a/b
MT	40.17 ± 0.01^i^	−14.28 ± 0.01^i^	27.86 ± 0.03^d^	−0.51 ± 0.00^j^
MY	43.62 ± 0.03^f^	−14.10 ± 0.01^h^	29.74 ± 0.39^a^	−0.47 ± 0.01^f^
ML	46.28 ± 0.06^e^	−13.09 ± 0.04^g^	28.90 ± 0.03^b^	−0.45 ± 0.00^d^
MR	46.43 ± 0.01^d^	−13.12 ± 0.02^g^	28.39 ± 0.01^c^	−0.46 ± 0.00^e^
MJ	42.61 ± 0.01^g^	−12.20 ± 0.02^d^	26.76 ± 0.01^e^	−0.46 ± 0.00^d^
GM1	46.79 ± 0.06^c^	−14.13 ± 0.16^h^	29.57 ± 0.36^a^	−0.48 ± 0.00^g^
GM2	49.50 ± 0.02^a^	−12.06 ± 0.01^c^	28.18 ± 0.05^c^	−0.43 ± 0.00^c^
ZM1	37.05 ± 0.02^k^	−12.68 ± 0.01^e^	25.15 ± 0.02^g^	−0.50 ± 0.00^i^
ZM2	37.93 ± 0.03^j^	−8.30 ± 0.03^a^	22.39 ± 0.03^h^	−0.37 ± 0.00^a^
JM	47.04 ± 0.01^b^	−11.17 ± 0.02^b^	26.60 ± 0.02^e^^f^	−0.42 ± 0.00^b^
M-GS	41.52 ± 0.01^h^	−12.96 ± 0.02^f^	26.44 ± 0.02^f^	−0.49 ± 0.00^h^

Data are shown as the mean ± standard deviation (*n* = 3). Different letters in each column represent significant difference (*p* < 0.05).

To sum up, the particle size of matcha in different regions is small, meeting the requirements of the national standard (≤18 μm). In addition, combined with the results of color difference measurement value and chlorophyll content, we can judge that MT, MY, GM1, ZM1 and M-GS have high green degree and good appearance quality.

### Analysis of main flavor substances of matcha of different regions

The quality components, tea polyphenols, soluble sugars, caffeine, L-theanine and free amino acids are usually used to evaluate the taste of tea products (28). These substances in the samples of matcha are displayed (Table 3). Free amino acids are important components affecting the fresh taste of tea soup (29). The content of all samples was relatively high, ranging from 1.43% to 4.07%, with an average value of 2.40%. L-theanine is the highest content of amino acid in matcha, which contributes to the fresh taste of tea soup. The results showed that the content of L-theanine in tea samples was 9.30 ~ 28.51 mg/g. Tea polyphenols are related to bitter and astringent of tea soup. The content of tea polyphenols in tea samples was 13.58 ~ 20.56%, with an average of 16.19%. Caffeine is highly related to the bitter taste of tea soup. The content of caffeine in tea samples was 1.72 ~ 4.41%, with an average of 3.26%. The soluble sugar content was 11.94 ~ 20.29 mg/g, and the average content was 16.04 mg/g. It was reported that the soluble sugar could reduce the bitter taste in the tea soup, and had positively effects on the sweet, mellow and thickness of the tea soup. In addition, the ratio of tea polyphenols to amino acid (RTA) can better reflect the contrast relationship between the strength and freshness of the taste of tea (29). The RTA valuesof the samples was 3.46 ~ 13.85, and 9 of 11 samples of matcha was less than 10. The taste of matcha requires fresh and mellow, and the low RTA values is good for its quality. In conclusion, matcha from different regions has the characteristics of low content of tea polyphenols, high content of free amino acids and L-theanine, and fresh taste. These components make synergistic contribution to the taste quality of matcha.

**Table 3:** The content of basic taste components of matcha from different regions.

Samples	Tea polyphenols (%)	L-Theanine (mg/g)	Free amino acid (%)	Caffeine (%)	Soluble sugar (mg/g)	RTA
MT	14.09 ± 0.05^e^	28.51 ± 0.44^a^	4.07 ± 0.32^a^	2.40 ± 0.27^e^	13.47 ± 0.25^de^	3.46
MY	13.58 ± 0.17^e^	25.02 ± 1.05^b^	3.57 ± 0.06^b^	3.69 ± 0.05^c^	13.75 ± 1.34^de^	3.80
ML	16.98 ± 0.21^c^	16.80 ± 0.35^c^	2.44 ± 0.26^c^	4.41 ± 0.18^a^	18.45 ± 2.24^abc^	6.96
MR	14.19 ± 0.26^e^	16.67 ± 0.13^c^	2.38 ± 0.25^c^	4.30 ± 0.09^a^	18.86 ± 0.60^ab^	5.96
MJ	14.29 ± 0.17^e^	17.00 ± 1.04^c^	2.42 ± 0.16 ^c^	4.34 ± 0.05^a^	17.99 ± 0.87^bc^	5.90
GM1	20.56 ± 0.09^a^	16.12 ± 0.16^c^	2.29 ± 0.17^cd^	3.87 ± 0.09^bc^	14.87 ± 1.03^d^	8.98
GM2	17.12 ± 0.56^c^	13.16 ± 0.31^d^	1.89 ± 0.34^e^	3.99 ± 0.12^b^	15.07 ± 0.27^d^	9.05
ZM1	15.57 ± 0.10^d^	15.65 ± 0.47^c^	2.24 ± 0.34^cd^	2.09 ± 0.02^f^	14.90 ± 0.51^d^	6.95
ZM2	15.60 ± 0.70^d^	10.25 ± 0.43^e^	1.47 ± 0.39^f^	1.72 ± 0.01^g^	16.83 ± 1.00^c^	10.61
JM	19.81 ± 0.19^b^	9.30 ± 0.18^ef^	1.43 ± 0.10^f^	3.00 ± 0.11^d^	20.29 ± 1.16^a^	13.85
M-GS	16.31 ± 0.26^d^	15.14 ± 0.48^c^	2.18 ± 0.07^d^	2.10 ± 0.03^f^	11.94 ± 0.26^e^	7.48

Data are shown as the mean ± standard deviation (*n* = 3). Different letters in each column represent significant difference (*p* < 0.05).

### Identification and quantitative analysis of volatile components in matcha from different regions

#### Identification and quantitative analysis of volatile components in matcha from different regions by headspace solid phase microextraction/GC–MS

**Figure 2:**
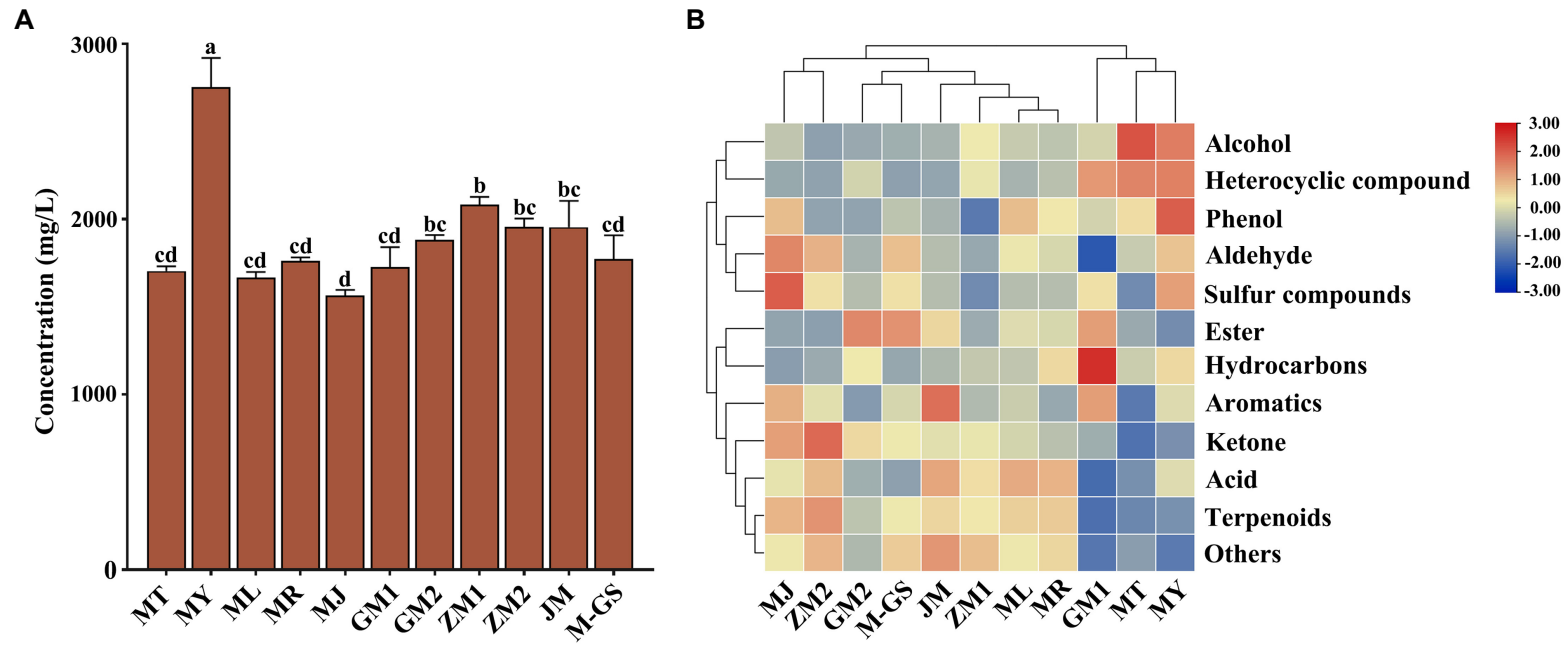
Analysis of concentration column chart and cluster heat map of total volatile components in matcha samples detected by HS-SPME. **(A)** Column chart of total volatile component concentration in tea sample. **(B)** Cluster heat map analysis of different volatile components in matcha samples. The lowercase alphabetical letters on the column chart represent significant differences (*p*<0.05).

A total of 534 volatile components were identified by HS-SPME. The concentration of total volatile components was 1563.59 ~ 2754.09 mg/L (Supplementary Table S1), among which MY (2754.09 ± 287.62 mg/L) was the highest, followed by ZM1, ZM2 and JM, while MJ was the lowest (Figure 2A). The concentration of total volatile components in Japanese matcha (MT, MY, ML, MR, and MJ) was lower than that in matcha from other regions except MY. We divided these volatile compounds into 12 different groups based on their chemical structure, including 139 terpenoids, 87 esters, 36 aromatics, 8 phenols, 36 alcohols, 61 ketones, 26 hydrocarbons, 31 aldehydes, 23 acids, 24 amines, 51 heterocyclic compounds and 12 other groups. The mean values of relative concentrations of different volatile components and their proportions in total concentrations have been tabulated (Supplementary Table S1). Among the 534 compounds, a total of 135 substances (mainly composed of esters and terpenoids) had higher concentrations (>1 mg/L) in all tea samples (Supplementary Table S2). By difference analysis (VIP > 1, *p* < 0.05), 78 compounds were screened out with no significant differences. The results showed that these compounds were the main components of volatile compounds in matcha, such as 2-decen-1-ol, (E)-, (E) -2-decenal, β-ionone epoxide, geraniol, (Z) -linalool oxide (pyranoid). The contribution of aroma compounds to the overall flavor of matcha depends not only on the concentration of the compounds in the tea soup, but also on the threshold value, which is an important reference standard. In order to evaluate the impact of the high concentration substances common to the overall flavor of matcha in different regions, we screened six key aroma components by calculating the OAV value, which may have an important contribution to the aroma quality of “seaweed-like” of matcha (Supplementary Table S3), such as (E)-2-decadienal, linalyl acetate and geraniol. While 57 compounds such as 1-hexadecanol and methyl jasmonate were significantly different, which may contribute to the formation of different aroma characteristics of matcha.

In order to more directly reflect the distribution of volatile components in matcha in different regions, we made a cluster heat map based on the data of the proportion of total concentration of each volatile component in tea samples (Figure 2B). As shown in the figure, tea samples were divided into four groups by clustering. As shown in the Figure 2B, the proportion of aldehydes, ketones and terpenoids in Group I (MJ, ZM2) was higher. The proportion of acids, terpenoids and ketones in Group II (GM2, M-GS, JM, ZM1, ML, and MR) was higher. Group III was GM1, which had the highest proportion of hydrocarbons, heterocyclic compounds, esters and aromatics among all the tea samples. Group IV (MT, MY) was characterized by high proportion of alcohols, heterocyclic compounds and esters.

#### Identification and quantitative analysis of volatile components in matcha from different regions by SAFE/GC–MS

**Figure 3:**
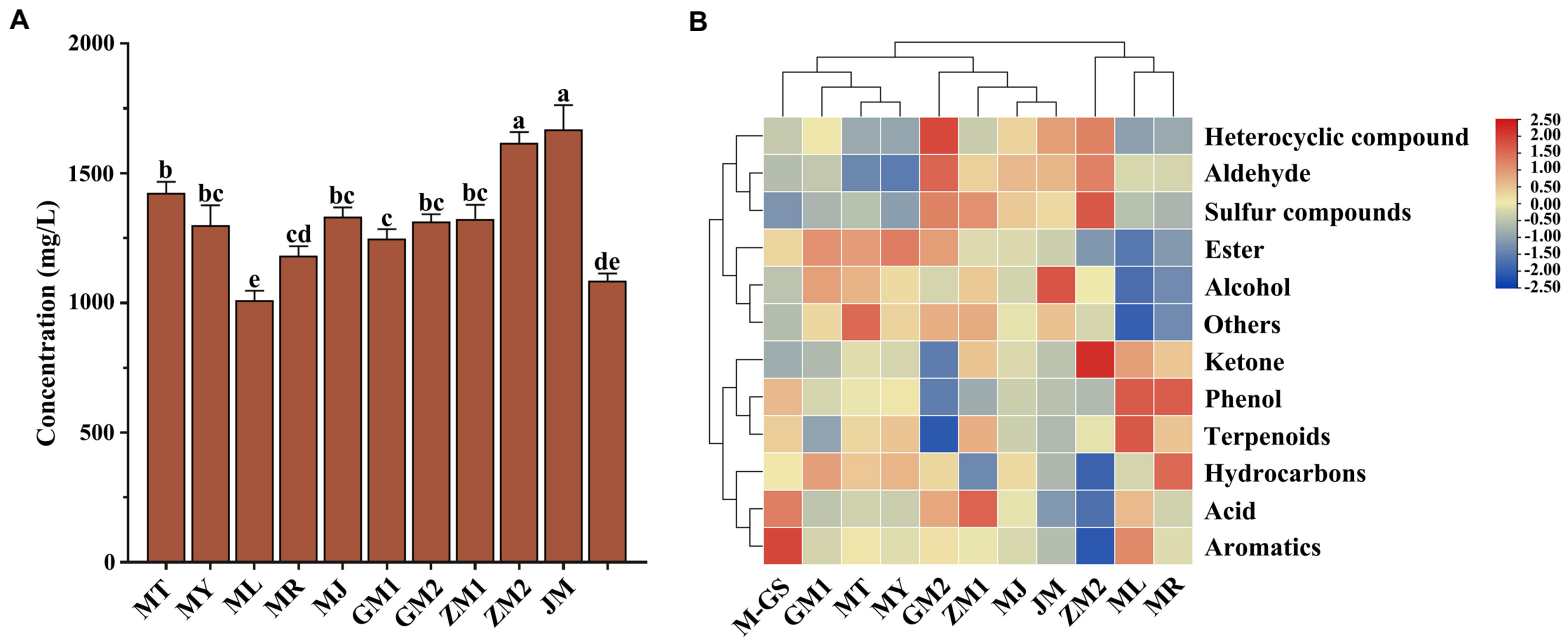
Analysis of concentration column chart and cluster heat map of total volatile components in matcha samples detected by SAFE. **(A)** Column chart of total volatile component concentration in tea sample. **(B)** Cluster heat map analysis of different volatile components in matcha samples. The lowercase alphabetical letters on the column chart represent significant differences (*p*<0.05).

A total of 1,097 volatile components were identified by SAFE, and the concentrations of these volatile components was 1009.21 ~ 1661.98 mg/L (Supplementary Table S1). Among all tea samples, the concentration of JM (1661.98 ± 64.66 mg/L) was the highest, followed by ZM2 (1616.58 ± 72.94 mg/L) and MT (1425.08 ± 73.02 mg/L; Figure 3A). These volatile substances were classified into 12 groups according to their chemical structure, including 23 amines, 209 terpenoids, 163 esters, 199 heterocyclic compounds, 72 aldehydes, 83 ketones, 31 phenols, 83 hydrocarbons, 70 aromatics, 41 other classes, 35 acids, and 88 alcohols. Among the 1,097 compounds, 120 volatiles (mainly composed of terpenoids, esters, heterocyclic compounds and hydrocarbons) had higher concentrations (>1 mg/L) in all tea samples (Supplementary Table S2). By difference analysis, 69 compounds with no significant differences were screened out, such as benzyl alcohol, 3-hexen-1-ol, (Z)-, benzaldehyde, naphthalene, these compounds are the main components of the volatile components of matcha. As in section 2.3.2, eight key aroma components were identified by calculating the OAV value of high concentration substances (Supplementary Table S3), such as 3-hexen-1-ol, (Z) -, 2-penten-1-ol, (Z) -, benzaldehyde, benzeneacetaldehyde. However, 51 compounds such as indole and limonene had significant differences, which may be the main substances causing different aroma characteristics of matcha in different regions.

Tea samples were divided into four groups through cluster analysis (Figure 3B). As shown in the Figure 3B, Group I included M-GS, GM1, MT, and MY, the proportion of esters and hydrocarbons in this group was higher, among which M-GS contained a high proportion of aromatics. Group II included GM2, ZM1, MJ and JM, and the proportion of tea aldehydes, heterocyclic compounds and sulfur compounds was relatively high, while the proportion of phenols was the lowest. Group III was ZM2, which had the highest proportion of ketones among all tea samples, and characterized by high proportion of heterocyclic compounds, aldehydes and sulfur compounds. Group IV included ML and MR, and the tea samples in this group characterized by high proportion of ketones, phenols and terpenoids.

### Analysis of volatile constituents in matcha from different regions

#### Principal component analysis of volatile components in matcha from different regions

**Figure 4:**
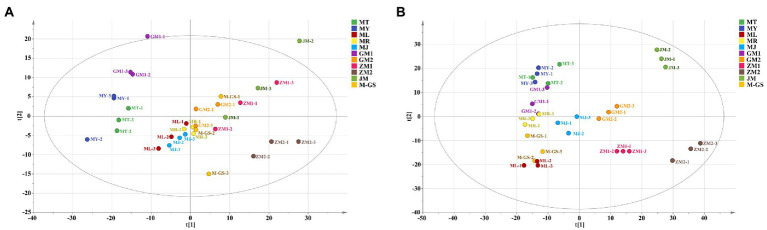
Principal component analysis of volatile components of matcha in different regions. **(A)** Principal component analysis of volatile components of matcha in different regions in HS-SPME. **(B)** Principal component analysis of volatile components of matcha from different regions in SAFE.

Principal component analysis is a statistical method that simplifies the data and interprets most of the original information by converting multiple variables into several composite variables through dimensionality reduction. This method is usually used to explore the comprehensive variables between samples and interpret the information in them (30, 31). PCA has been widely used to study the volatile components of tea (30). In this study, the GC–MS analysis results of volatile components of matcha from 11 different regions were used for principal component analysis. The PCA results of HS-SPME (Figure 4A) showed that the contribution rates of PC1 and PC2 are 38 and 12% respectively, and the total contribution rate is 50%. In the figure, MT, MY, and GM1 are closer together and can be divided into one category, the second category include ML, MR, MJ, ZM1, M-GS and JM, and the third category is ZM2. Three groups of tea samples can achieve better differentiation, the difference is more obvious. The PCA results of the SAFE (Figure 4B) showed that the contribution rates of PC1 and PC2 are 28.6 and 19.8% respectively, and the total contribution rate is 48.4%. JM and ZM2 are distributed on the right side of the figure, MJ, ZM1, and GM2 are distributed in the middle of the figure, and the rest of the tea samples were distributed on the left side of the figure, the distance is relatively close, and the degree of differentiation is low. On the whole, PCA results showed that the distinguishing effect is poor among tea samples is low, which cannot clearly reflect the differences between samples. Therefore, we will explore the differences and characteristics of volatile components among tea samples through the next step of cluster analysis.

#### Cluster analysis of volatile components of matcha from different regions

**Figure 5:**
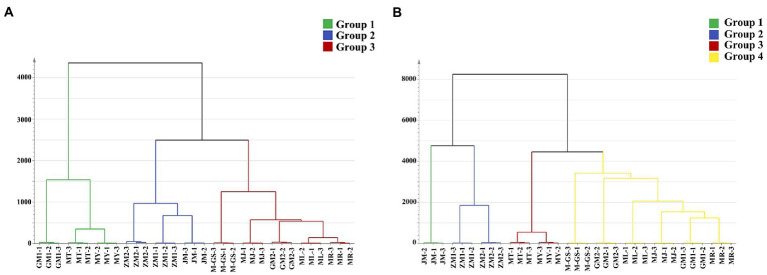
Cluster analysis tree diagram of matcha in different regions. **(A)** HS-SPME. **(B)** SAFE.

Hierarchical cluster analysis (HCA) is widely used to evaluate the volatile components of tea (32, 33). In this study, HCA was used to analyze the similarity of tea samples, and then tea samples were grouped. OPLS-DA analysis was used to calculate the contribution score (contribution score > 0) of volatile components in each grouped tea sample, which was positively correlated with its aroma. Based on this, the contribution score of a single substance in different groups is compared, and calculated the VIP value (VIP > 1) for the higher contribution score is determined as the characteristic aroma component of this group of tea samples. As can be seen from the tree diagram of volatile components of samples detected by HS-SPME (Figure 5A), tea samples were divided into three groups. Samples in the same group had high similarity, while samples in different groups would be vary greatly. Group I was composed of GM1, MT and MY, OPLS-DA analysis showed that 120 compounds are positively correlated with aroma quality of tea samples (Supplementary Table S4) Based on VIP values, 45 components such as decanal (sweet, waxy, citrus, floral), pyrazine, 3,5-diethyl-2-methyl- (nutty, meaty, vegetable), 1-hexadecanol (waxy, clean, floral) were identified as the key difference compounds between this group and other groups, that is, they are the characteristic aroma components of this group, which contributed to the formation of aroma. Group II was composed of ZM1, ZM2 and JM, with a total of 441 substances positively correlated with its aroma quality (Supplementary Table S4). After screening, a total of 225 compounds were its characteristic aroma components, such as coumarin (tonka, sweet), furan, 2-pentyl—(fruity, green, earthy, beany, metallic), dihydro-β-ionone (earthy, woody, orris, dry). Group III included ML, MR, MJ, GM2 and M-GS, 192 substances were positively correlated with aroma quality in this group of tea samples (Supplementary Table S4). A total of 15 compounds were selected as the characteristic aroma components, including γ-terpinene (woody, terpenic, lemon, herbal), myrtenol (herbal, woody, balsamic, sweet), *cis*-3-hexenyl valerate (green, fruity) and so on.

The 11 tea samples detected by SAFE were divided into four groups (Figure 5B), group I was JM, and the results of OPLS-DA analysis showed that 900 compounds (Supplementary Table S5) are positively correlate with their aroma quality. Through the screening of VIP values, 555 components (Supplementary Table S5), such as benzyl alcohol (floral, phenolic, balsamic), cyclopentanone, 2-(2-hexyl)- (floral), geranic acid (waxy), 1-hexanol (herbal, fruity, sweet, green), were considered as their characteristic aroma components, these compounds have the aroma characteristics of green, sweet, fruity and floral, which were conducive to the formation of JM aroma. Group II included ZM1 and ZM2, 581 compounds were positively correlated with their aroma quality (Supplementary Table S5) in this group. After screening, 171 compounds were characteristic aroma components, contributing to the formation of tea-like green and floral aroma in this group, such as hexanal (fresh, green, fatty, aldehydic, grassy, leafy, fruity, sweat), dimethyl trisulfide (sulfurous, onion, cooked, onion), hexanoic acid, methyl ester (fruity). Group III included MT, MY, a total of 562 compounds were positively correlated with herbal aroma (Supplementary Table S5), and the 178 substances such as 2-pentanol (green, fusel, oily, fermented) were the characteristic aroma components, which can contribute to the formation of green, sweet and herbal aroma, such as safranal (herbal), geraniol (sweet, floral, fruity), 3-hexen-1-ol, (Z)- (green). The Group IV, including ML, MR, MJ, GM1, GM2 and M-GS, had a total of 352 substances that were positively correlated with their aroma, with few characteristic components, which were isocyclocitral (green, aldehydic, earthy, woody, herbal, and leaf), 4-hydroxy-2-methylacetophenone.

In conclusion, according to the clustering of volatile components, Japanese matcha was mainly divided into two groups. Class I (MT, MY) has a higher level, with a total of 212 characteristic aroma components. Class II (ML, MR, and MJ) had high similarity in aroma characteristics, and 194 compounds were the characteristic aroma components. The overall aroma characteristics of GM1 were similar to those of Japanese matcha, with 47 aroma components. There were 395 characteristic aroma components in Zhejiang matcha (ZM1, ZM2), and JM also displays similar aroma characteristics with Zhejiang matcha due to 225 components. In addition, JM also has unique aroma characteristics, mainly relates to 555 components. M-GS, GM2 and Japanese matcha (ML, MR, and MJ) have similar aroma characteristics.

## Discussion

The quality of matcha is mainly evaluated from three aspects: sight, taste and smell. According to the results of color difference measurement value and chlorophyll content of tea samples, the green degree and the content of chlorophyll in all samples were high, and fresh green color. For the chlorophyll content of matcha, it was described in the literature as high as 17 mg/g (34), while the content of green tea was 1 ~ 10 mg/g (34, 35). In any case, the chlorophyll content of matcha in different regions was at a high level, and the ratio of Chla/Chlb was low, which was due to the unique shading technology of matcha. The fresh tea leaves grown under shading conditions contains higher total chlorophyll content than the tea processed by other technologies (36, 37). The Chla/Chlb can be used to evaluate the quality of matcha (27), and the tea samples with low ratio have higher quality. It is well known that light-harvesting complex (LHC) was rich in Chlb (38), and under shading conditions, plants need to capture as much light as possible to increase their LHC size and thus synthesize more Chlb. Therefore, tea plants growing under low light conditions will promote the synthesis of Chlb, resulting in a lower Chla/Chlb ratio.

Tea polyphenols are important taste substances in tea and play a unique role in the sensory quality of tea, including color, bitterness and astringency (39). The free amino acids are conductive to mellow and umami flavor, which can be used as a good indicator to evaluate the quality of tea. Caffeine is the main purine alkaloid with a bitter taste in tea (40). High-quality matcha has the characteristics of high content of chlorophyll, amino acid and protein, and low content of tea polyphenols and caffeine. The taste quality of MT and MY in Japanese matcha is better than the other three Japanese matcha, and the taste is more fresh and mellow; The freshness of GM2, ZM2 and JM is insufficient, which is related to the lower amino acid content; The taste of GM1, ZM1 and M-GS is also relatively harmonious, similar to that of Japanese matcha ML, MR and MJ.

The volatile components in tea samples can be fully extracted by HS-SPME and SAFE procedure. The amount of volatile substances extracted by SAFE was more than by HS-SPME, because the SAFE dissolves the volatile substances in tea samples in organic solvents, the extraction method was mild and the loss of heat sensitive volatile substances was less, which can better reduce the natural flavor of tea samples (41). In addition, the two methods have different extraction effects on different kinds of volatile compounds, the high proportion of terpenoids, esters and alcohols extracted by HS-SPME was due to the terpenoids have good aroma and generally high boiling point (42), while the temperature required by HS-SPME technique was higher than SAFE. Contrary to HS-SPME, SAFE can extract a higher proportion of heterocyclic compounds, sulfur compounds, hydrocarbons, ketones and phenols from tea samples. It has been reported that the sulfur compounds in tea samples cannot be absorbed by HS-SPME coating materials (43), and the extraction efficiency of SAFE for sulfur compounds is higher (44).

The substances with high concentration and no difference between samples constitute the common components of the aroma of matcha. Among the substances detected by HS-SPME, 2-decen-1-ol, (E)- and (E)-2-decenal contribute to the formation of black tea aroma (45). β-ionone epoxide was a carotenoid derivative, and the shading technology of matcha can increase the content of carotenoids, thus increasing the content of derivative aromatic compounds (13). Geraniol, a typical enol aroma compound in tea, has been proved to be released from its glycoside aroma precursor through the action of endogenous glycosides (46), with a pleasant rose aroma (47), which often appears in green tea and had a high content in Longjing tea (48), has been reported to be an important volatile compounds of black tea, yellow tea and oolong tea (49–51), which was a key indicator of tea flavor quality (52, 53), and has a high content in matcha. It shows that it plays an important role in the aroma quality of matcha in different regions. Among the substances identified by SAFE, 3-hexen-1-ol, (Z)- has been reported to be the most dominant source of green aroma in green tea (54). In aromatic aldehyde, benzaldehyde contributes to the formation of the aroma of chestnut green tea (55), with a strong almond-like and fruity aroma (56). Naphthalene was a key aroma component of Chinese yellow tea (51), which contributed to the formation of green tea aroma. These compounds were the main components of matcha volatilities due to their high concentrations in tea samples, played an important role in the aroma quality of matchaand contributed to matcha aroma formation. OAV value is usually used to characterize the contribution of individual compounds to aroma. Among the substances with high OAV value identified in this study, the threshold of (E)-2-decenal is very low, and has the aroma characteristics of waxy, fatty, earth and green. Benzeneacetaldehyde was the main aroma component of black tea (57), showing pleasant aroma, with green, sweet, and floral aroma properties. Ethylbenzene was the key aroma component of chestnut green tea (55). It is worth mentioning that the thresholds of 14 key aroma components vary greatly, among which, (E)-2-decental (waxy, fatty, earthy, green), geraniol (sweet, floral, fruity), benzeneacetaldehyde (green, sweet, floral), ethylbenzene, 1,3-benzodioxole, 4-methoxy-6-(2-propyl) -, hydroxymethyl cyclopentanone (caramellic), maple furanone (sweet, fruity, caramellic), has a low threshold and a large contribution. The threshold value of naphthalene (pungent, dry, resinous)，3-hexen-1-ol, (Z)- (green), toluene (sweet), benzaldehyde (fruity), δ-dodecalcactone (fresh, sweet, metallic), linalyl acetate (herbal, sweet, green, citrus), 2-penten-1-ol, (Z)- (green, phenolic, fruity) is relatively high, but they all have certain contributions. These substances play different roles, coordinate in a certain proportion, and participate in the formation of the special flavor type of “seaweed-like” aroma.

The volatile components with high concentration and significant difference in the samples are the reasons for the different aroma characteristics of matcha. For example, methal jasmonate was an important fatty acid derivative, which was usually reported in oolong tea and was the main source of flower aroma (58). Limonene was a key aroma compounds in Keemun, Assam, Darjeeling and Ceylon black teas (57). Heterocyclic compounds were mainly derived from the maillard reaction, and the products generally have the aroma characteristics of roasted and nutty. Indole was the characteristic aroma components of oolong tea (59), it was reported that the diluted indole has sweet taste and flower aroma, and in a specific content range to enhance the overall flavor of green tea (60). It is worth noting that the differences in these volatile components can be attributed to the differences in tea varieties, such as the differences in the phytochemical characteristics of fresh tea leaves, processing parameters and environmental conditions due to the differences in tea varieties planted. In addition, the formation of tea aroma generally comes from the tea raw material itself and the tea processing process, such as lipid degradation, maillard reaction, carotenoid degradation and so on (17), which may lead to differences in the aroma. This part was based on the analysis of the high concentration aroma components of the tea samples from different regions. Therefore, in order to clearly distinguish the aroma characteristics of the tea samples from different regions, a follow-up study has been carried out.

Hierarchical cluster analysis grouped tea samples with similar aroma characteristics, and Japanese matcha was grouped into Class I (MT, MY) and Class II (ML, MR, MJ). Among the characteristic components of Class I matcha, (E,E)-2,4-nonadienal was a representative key aroma component in green tea (61), and also an important contributor to the aged fragrance of Qingzhuan tea (62), which has been reported to be the main aroma component of Japanese matcha (11) 2-acetylthiazole has the aroma characteristics of nutty, grain-like and popcorn-like aroma with low odor threshold (63). (E)-nerolidol was a volatile sesquiterpene, which contributes to the formation of floral aroma and is an important aroma component of oolong tea (64). It also contributes to the formation of chestnut aroma of green tea (55). The characteristic aroma components of Class II were less. We speculate that the concentration and proportion of volatile components of Class II were different from that of Class I, and the aroma intensity of tea samples was lower than that of Class I, while these compounds have a higher contribution in Class I. The aroma characteristics of Guizhou matcha (GM1, GM2) and Japanese matcha were similar. GM1 was mainly clustered with Japanese matcha of Class I. Among the functional characteristic components, hexadecanol acid was reported to contribute significantly to the unique aroma of oolong tea (65), and 1-hexadecanol constitutes the aroma quality of white tea (66). GM1, GM2 and M-GS have similar aroma characteristics to the matcha of Class II, and among the substances that are positively correlated with their aroma quality, 2, 6-nonadienal, (E,Z)- (green, fatty, dry, cucumber), 4-heptenal, (Z)-(green), 2, 4-heptadien-1-ol, (E,E)-(fatty, green, fruity), p-cresol (phenolic, narcissus, animal, mimosa), pyrazine, 2-ethyl-3, 5-dimethyl- (nutty, burnt, almond, coffee) and other substances have been reported as key aroma components in Japanese matcha tea (11). Among the characteristic aroma components, γ-terpinene was the marker compound of aroma difference in different fermentation stages of Pu ‘er tea (67). It is reported that Guicha Group, which produces Guizhou matcha, has hired senior Japanese matcha engineers to guide matcha production from variety selection, cultivation management to product processing. This may be the reason why the aroma quality of Guizhou matcha is similar to that of Japanese matcha. The tea areas in Shandong, China, especially Rizhao and Qingdao, are close to the latitude of Shizuoka, Japan, and are also affected by the marine climate. In addition, the complete set of matcha processing and production lines of Shandong matcha were introduced from Japan. These should be the reason why the aroma quality of Shandong matcha is similar to that of Japanese matcha.

Among the characteristic volatile components of Zhejiang matcha (ZM1, ZM2), hexanal has the characteristics of seaweed-like aroma, which has been proved to contribute to the formation of seafood aroma (68), and was the key aroma component of Japanese matcha (11, 13), as well as the characteristic aroma component of Fu Zhuan tea (69). Among the characteristic volatile components of JM, benzyl alcohol was a glycoside derivative (70), with the aroma characteristics of floral, phenolic and balsamic. Among the characteristic components that lead to the similar aroma characteristics of Zhejiang matcha and Jiangsu matcha, cedrol has been reported to be the main aroma component of black tea (71), which had an important contribution to the woody aroma of Fu Brick tea (16). Furan, 2-pentyl- has seaweed characteristics and has been shown to contribute to the formation of seafood aroma (68), plays an important role in the aroma formation of Japanese matcha (13), and was also a major contributor to the roasted aroma of white tea (16). Coumarin has been identified as a key odorant in green tea due to its high FD factor (64–4,096) (61), which mainly exists in free form in fresh tea leaves. Both steaming time and drying temperature affect the concentration of coumarin in the final green tea product (72). In Japanese green tea, the content of coumarin was 0.26 ~ 0.88 μg/g (72). The relatively long brewing time and low soaking temperature contribute to the aroma of pleasant sweet-herbaceous and cherry flower-like odor of green tea soup. Cubenol can provide clean and fresh aroma. According to the study of Jiang SHI et al., exogenous methyl jasmonate treatment can induce the production of cubenol when discussing the influence of the treatment on tea aroma (73). The reason why the volatile components of Jiangsu matcha and Zhejiang matcha, especially Jiangsu matcha, are different from those of other regions is that the raw materials used to produce Jiangsu matcha are hot air fixation instead of steam fixation. It has been reported that hot air fixation can help to produce strong and lasting flower and chestnut fragrance (74).

## Conclusion

The application of various grinding methods such as air mill, ball mill and stone mill in the production of matcha has made the particle size of matcha uniform and fine. The particle size of the 11 tested samples of matcha is basically controlled with 10 μm, with soft and uniform characteristics. The commonly used shading technology has improved the chlorophyll content in matcha, decreased the phenol-ammonia ratio, and the taste is more fresh and mellow. The test samples selected in this study have performed better overall, especially Guizhou matcha (GM1), Zhejiang matcha (ZM1), and Shandong matcha (M-GS). The substances with high concentration in all samples were the common components of the aroma of matcha, and 14 compounds that may contribute to the formation of “seaweed-like aroma” of matcha have been identified through OAVs calculation. The different volatile components caused different aroma characteristics among different regions of matcha samples. In our study, Guizhou matcha (GM1, GM2) and Japanese matcha (MT, MY, ML, MR, and MJ) have similar aroma characteristics, Zhejiang matcha (ZM1, ZM2) and Jiangsu matcha (JM) have similar aroma characteristics, and each has its own unique flavor, Shandong matcha (M-GS) and Class II Japanese matcha (ML, MR, MJ) have similar aroma characteristics, and OPLS-DA analysis was used to identify the characteristic aroma components of different geographical groups of matcha. This study clarified the material basis for the flavor differences of matcha in different regions in the Chinese market, and the research results can provide a theoretical basis for the selection and application of matcha in drinks and tea products.

## Data availability statement

The original contributions presented in the study are included in the article/Supplementary material, further inquiries can be directed to the corresponding author.

## Author contributions

YL: conceptualization, data curation, formal analysis, methodology, software, and writing—original draft. YZ: data curation, formal analysis, software, and writing–review and editing. FQ, WQ, and PW: data curation, formal analysis, and software. XZZ: resources and validation. XFZ: funding acquisition and supervision. JH: conceptualization, funding acquisition, methodology, validation, supervision, and writing—review and editing. All authors contributed to the article and approved the submitted version.

## Funding

This research was funded by the Natural Science Fund of Shandong Province (No. ZR2019MC039), National Natural Science Foundation of China (Grant No. 32272767), Youth Innovation and Science Technology Support Program of Shandong Province (No. 2021KJ103), Project of Laoshan District Tea Innovation Group (No. LSCG2022000017), and Qingdao West Coast New Area Science and Technology Plan Source Innovation Cultivation Special Project (No. 2020–96).

## Conflict of interest

The authors declare that the research was conducted in the absence of any commercial or financial relationships that could be construed as a potential conflict of interest.

## Publisher’s note

All claims expressed in this article are solely those of the authors and do not necessarily represent those of their affiliated organizations, or those of the publisher, the editors and the reviewers. Any product that may be evaluated in this article, or claim that may be made by its manufacturer, is not guaranteed or endorsed by the publisher.

## Footnotes

^1^https://cloud.metware.cn/
